# LINE-1 methylation shows little intra-patient heterogeneity in primary and synchronous metastatic colorectal cancer

**DOI:** 10.1186/1471-2407-12-574

**Published:** 2012-12-05

**Authors:** Aika Matsunoki, Kazuyuki Kawakami, Masanori Kotake, Mami Kaneko, Hirotaka Kitamura, Akishi Ooi, Go Watanabe, Toshinari Minamoto

**Affiliations:** 1 Division of Translational and Clinical Oncology, Cancer Research Institute, Kanazawa University, 13-1 Takara-machi, Kanazawa, 920-0934, Japan; 2 Department of General and Cardiothoracic Surgery, Kanazawa University Graduate School of Medical Science, 13-1 Takara-machi, Kanazawa, 920-8641, Japan; 3 Department of Molecular and Cellular Pathology, Kanazawa University Graduate School of Medical Science, 13-1 Takara-machi, Kanazawa, 920-8641, Japan

**Keywords:** LINE-1, DNA methylation, Colorectal cancer, Laser microdissection

## Abstract

**Background:**

Long interspersed nucleotide element 1 (LINE-1) hypomethylation is suggested to play a role in the progression of colorectal cancer (CRC). To assess intra-patient heterogeneity of LINE-1 methylation in CRC and to understand its biological relevance in invasion and metastasis, we evaluated the LINE-1 methylation at multiple tumor sites. In addition, the influence of stromal cell content on the measurement of LINE-1 methylation in tumor tissue was analyzed.

**Methods:**

Formalin-fixed paraffin-embedded primary tumor tissue was obtained from 48 CRC patients. Matched adjacent normal colon tissue, lymph node metastases and distant metastases were obtained from 12, 18 and 7 of these patients, respectively. Three different areas were microdissected from each primary tumor and included the tumor center and invasive front. Normal mucosal and stromal cells were also microdissected for comparison with the tumor cells. The microdissected samples were compared in LINE-1 methylation level measured by multicolor MethyLight assay. The assay results were also compared between microdissected and macrodissected tissue samples.

**Results:**

LINE-1 methylation within primary tumors showed no significant intra-tumoral heterogeneity, with the tumor center and invasive front showing identical methylation levels. Moreover, no difference in LINE-1 methylation was observed between the primary tumor and lymph node and distant metastases from the same patient. Tumor cells showed significantly less LINE-1 methylation compared to adjacent stromal and normal mucosal epithelial cells. Consequently, LINE-1 methylation was significantly lower in microdissected samples compared to macrodissected samples. A trend for less LINE-1 methylation was also observed in more advanced stages of CRC.

**Conclusions:**

LINE-1 methylation shows little intra-patient tumor heterogeneity, indicating the suitability of its use for molecular diagnosis in CRC. The methylation is relatively stable during CRC progression, leading us to propose a new concept for the association between LINE-1 methylation and disease stage.

## Background

Global DNA hypomethylation is frequently observed in various malignancies including colorectal cancer (CRC) [1,2], where it is thought to play a pivotal role in carcinogenesis [3]. One of the possible mechanisms for the involvement of DNA hypomethylation in cancer development is through the activation of long interspersed nucleotide element-1 (LINE-1) and genomic instability [4,5]. LINE-1 is a non-long-terminal-repeat class of retroposon. It is the most successfully integrated mobile element and accounts for about 18% of the human genome [6]. LINE-1 has the potential to transpose in the human genome, thus creating new genetic sequences that are one of the driving forces of human evolution [7,8]. Although the majority of LINE-1-derived elements in the human genome no longer have the ability to transpose due to mutations and deletions in their sequence, approximately 100 full-length copies of LINE-1 retain this ability [9,10]. Global DNA hypomethylation is also accompanied by hypomethylation of LINE-1 promoter [11], suggested to result in aberrant expression and active transposition of this sequence. The hypomethylation and/or transposition of LINE-1 elements during carcinogenesis have been suggested to alter the transcriptome [12] and to play a role in the acquisition of multiple cancer phenotypes including invasion and metastasis.

Consistent with the suggested link between LINE-1 hypomethylation and carcinogenesis, previous studies reported that LINE-1 methylation levels are lower in more advanced stages of CRC, leading to the concept of a progressive loss of genomic methylation during CRC development [13]. The inverse association between LINE-1 methylation and CRC stage suggested that LINE-1 hypomethylation was causally involved in the acquisition of invasive and metastatic phenotypes since these are critical factors in TNM staging. If this was true, the LINE-1 methylation level could be expected to differ between the tumor center and invasive front, and between primary and metastatic tumor tissue from the same patient. However, previous studies have only measured LINE-1 methylation in the primary tumor and it is still unknown whether this differs from metastatic lesions.

Aside from its biological relevance, the possible heterogeneity of LINE-1 methylation is a potential problem for any clinical application using the methylation status. Molecular analyses of tumor samples are generally performed using biopsy specimens obtained prior to surgery, or using the surgically resected tissue. Because tissue from metastatic deposits is not easily accessible, results obtained from analysis of the primary tumor are used in clinical decision making. The strategies based on molecular analysis assume the marker shows no significant intra-patient heterogeneity. However, this issue is rarely investigated for candidate prognostic and predictive markers.

LINE-1 methylation is a promising prognostic factor in CRC [14] and may also be a predictive marker for the response to fluoropyrimidines in microsatellite stable and CpG island methylator phenotype-negative CRC [15]. For LINE-1 methylation to be used in personalized medicine, the intra-patient heterogeneity of this molecular marker first needs to be established. Furthermore, the impact of contamination of tumor tissue with stromal cells on the analysis of LINE-1 methylation also needs to be investigated. A previous study showed that stromal cells such as fibroblasts and infiltrating lymphocytes have significantly higher LINE-1 methylation levels compared to the adjacent tumor cells [13]. The degree of contamination of tumor specimens with stromal cells could therefore compromise the accuracy of LINE-1 methylation assays. Laser-capture microdissection (LCM) can reduce this interference by allowing the exclusive collection of tumor cells. However, LCM is labor intensive and time consuming compared to the use of whole tumor tissue for molecular diagnosis. Comparison of the results for LINE-1 methylation levels in microdissected tumors compared to whole tumors should resolve whether contamination with stromal cells has a significant influence on the measurement of this marker.

The issues of intra-patient heterogeneity and contamination with stromal cells were addressed in this study to increase our understanding of LINE-1 methylation in cancer biology, as well as for possible future clinical applications of this marker in CRC. Using LCM, CRC samples were collected from multiple sites including the center and invasive front of primary tumors, as well as from lymph node and distant metastases. LINE-1 methylation levels were found to show little intra-patient heterogeneity, indicating this marker is suitable for clinical applications. Stromal cells significantly influenced the measurement of LINE-1 methylation in tumors, demonstrating the need for LCM. Finally, from a biological perspective the prevailing view of a progressive loss of genomic methylation during CRC development was not supported in this study. Instead, we propose another mechanism to explain the link between LINE-1 methylation and disease stage in CRC.

## Materials and methods

### Patients and tissue samples

Formalin-fixed paraffin-embedded (FFPE) CRC tissue specimens were obtained from 48 patients who underwent surgery at Kanazawa University Hospital. The patients comprised 26 males and 22 females and ranged in age from 37–91 years (mean 68.8 years). In addition to the primary CRC tissue, FFPE specimens of matched adjacent normal colon tissue, lymph node metastases and distant metastases were available for 12, 18 and 7 of these patients, respectively. Tumor stage was defined according to the International Union Against Cancer (UICC) TNM system. Approval for this project was obtained from the Kanazawa University Medical Ethics Committee.

### Tissue collection by LCM and macrodissection

All tissue samples were reviewed for quality and tumor content, followed by histological diagnosis with hematoxylin-eosin staining. Ten μm thickness sections were placed on a special foil on a glass slide, deparaffinized with xylene and then hydrated and stained with hematoxylin. Cells were collected using the Leica AS LMD system (Leica mycrosystems, Wetzlar, Germany) and captured into a microcentrifuge tube. To verify the accuracy of capture, images of tissue sections taken before and after microdissection were recorded. Representative images were presented in Additional file 1: Figure S1. Cell populations from three different areas were collected and included the tumor center and invasive front for all 48 primary tumors. For 12 patients, matched epithelial cells from normal mucosa and stromal cells surrounding the tumor cells were also isolated. In 21 cases, metastatic tumor cells from lymph nodes and/or distant metastases were collected. In addition to the microdissected tissue samples, a scalpel was used to collect macrodissected tumor tissue for assessment of the influence of stromal cell contamination and for analysis of microsatellite instability (MSI) and CpG island methylator phenotype (CIMP) status.

### DNA isolation and bisulfite treatment

DNA was extracted from tissue samples using the QIAamp DNA FFPE tissue kit (Qiagen, Hilden, Germany) and treated with bisulfite using the EpiTect bisulfite Kit (Qiagen) according to the manufacturer’s protocols.

### Multicolor MethyLight assay

LINE-1 methylation was measured using the MethyLight assay [16] and the use of two different probes for unmethylated- and methylated-LINE-1 sequences. These were labelled with FAM and Yakima yellow, respectively. Primers used for PCR (forward, GGGAGTGTTAGATAGTGGG; reverse, AAACTCCCTAACCCCTTA) contained no CpG sites and amplifed the bisulfite-converted LINE-1 sequence independently of its methylation status. The two probes were synthesized by Nippon EGT (Toyama, Japan) and consisted of FAM-CCTACTTCAACTCACACACAATAC-Eclipse Dark Quencher and Yakima yellow-CCTACTTCGACTCGCGCACGATAC-Eclipse Dark Quencher. Locked nucleic acid was used for the underlined nucleotides in order to match the melting temperature between the probes. A standard sample was created by ligating unmethylated- and methylated-LINE-1 sequences, and cloning into a plasmid (Additional file 2: Figure S2). Real-time detection was performed simultaneously with the standard sample that was equivalent to 50% methylated LINE-1 sequence. The percentage of methylated LINE-1 was calculated using the formula: 100 × methylated reaction / (unmethylated reaction + methylated reaction).

### Analysis of MSI and CIMP

DNA isolated from macrodissected tissue was used for the analysis of MSI and CIMP as described previously [15]. MSI status was determined using 3 mononucleotide repeat markers (BAT26, NR21 and NR27) and CIMP by the methylation status of 5 genes (CACNA1G, IGF2, NEUROG1, RUNX3 and SOCS1).

### Statistical analysis

Paired t-test was used for the comparison between the LINE-1 methylation levels in different samples. The Mann–Whitney U test or the Kruskal-Wallis test was used to compare LINE-1 methylation levels between two or three clinicopathological variables, respectively. All statistical analyses were carried out using the R software package version 2.7.2 [17].

## Results

### Development and validation of a multicolor MethyLight assay

In a previous study we developed a quantitative methylation-specific PCR (qMSP) assay to measure LINE-1 methylation [18]. This assay used methylation-specific primers and SYBR green and was initially employed here to analyze the microdissected samples. However, the results obtained with this method were not reproducible due to the low threshold cycle (data not shown). We therefore developed a new multicolor MethyLight assay to measure LINE-1 methylation with high sensitivity and reproducibility. To validate this method, samples with 10% increments in LINE-1 methylation level were prepared by mixing varying ratios of plasmids cloned with unmethylated- or methylated-LINE-1 sequence. Using the multicolor MethyLight assay, a linear increase in LINE-1 methylation was observed in the prepared samples (Additional file 3: Figure S3). To evaluate inter-assay variation, DNA from four CRC cell lines with different LINE-1 methylation levels was analyzed in four independent assays. The inter-assay variation was within an acceptable range (Additional file 4: Table S1). These results demonstrate that the newly established assay was accurate and reproducible across a range of LINE-1 methylation levels.

### LINE-1 methylation level shows little intra-patient heterogeneity

**Figure 1:**
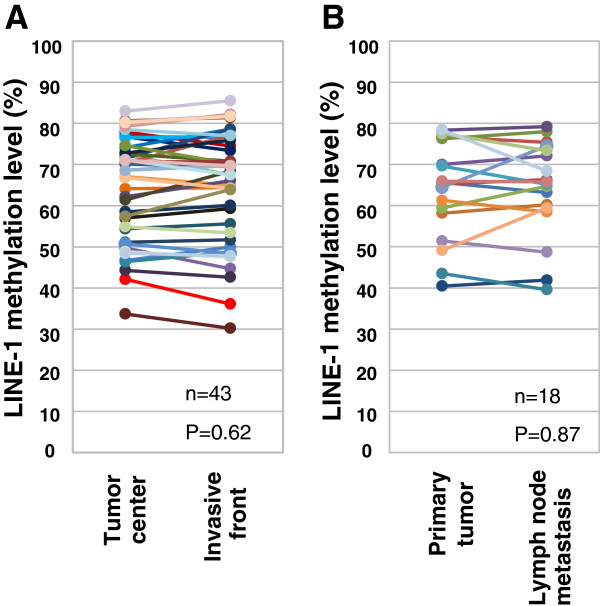
**LINE-1 methylation at multiple tumor sites.** LINE-1 methylation level was compared between cancer cells from the center and invasive front of the tumor **(A)**, and between the primary tumor and lymph node metastasis **(B)**.

Tumor samples microdissected from three different areas of each primary tumor including the center and invasive front were evaluated in duplicate by the multicolor MethyLight assay. Samples with a low threshold cycle value (<32) showed inconsistent results for duplicate assays and were thus deemed as “not available” (NA). All the results of the assay are shown in Additional file 5: Table S2. The measurement of LINE-1 methylation was successful in 133/144 (92.4%) of the microdissected samples from the primary tumors, despite the small amounts of DNA collected by LCM. The LINE-1 methylation level showed little intra-tumor heterogeneity, with all values from the same tumor being within 10% difference. This level of difference is not strong considering the distribution of the LINE-1 methylation (range of 27.2 to 88.3%, Additional file 5: Table S2) and up to 4.9% of the inter-assay variation (Additional file 4: Table S1). No significant difference was observed in LINE-1 methylation between the central area and invasive front from the same tumor (Figure 1A).

To investigate for possible differences in LINE-1 methylation between matched primary and metastatic tumor tissues, tumor cells collected by LCM from lymph node (18 cases) and distant metastases (7 cases) were also evaluated. Distant metastases from liver (5 samples), peritoneum (4 samples) and ovarium (1 sample) were available for the analysis. The mean LINE-1 methylation value observed in multiple areas of the primary tumor was identical to that found in metastatic lymph nodes (Figure 1B). No significant difference in the methylation between primary tumor and distant metastases was observed (Table 1). These results demonstrate there is little intra-patient heterogeneity for LINE-1 methylation and that it remains relatively stable during CRC progression.

**Table 1:** LINE-1 methylation level in the primary tumor and in synchronous metastases of CRC

**LINE-1 methylation level (%)**					
**Patient ID**	**Primary**	**Metastatic sites**			
		**Lymph node**	**Liver**	**Peritoneum**	**Ovarium**
12	73.4	NA	78.0	NA	NA
18	43.5	39.6	38.7	NA	NA
27	48.2	NA	NA	40.6	NA
29	59.5	64.7	66.9	65.2	NA
30	70.0	72.1	77.7	70.8	67.5
36	77.5	73.4	NA	74.6	NA
40	70.0	NA	66.6	NA	NA

The LINE-1 methylation level at the primary tumor site is the mean value of assay results from multiple areas including the tumor center and invasion front.

NA, samples were not available or no metastasis was found at the indicated site.

### Contamination of tumor sample with stromal cells significantly influences the measurement of LINE-1 methylation

**Figure 2:**
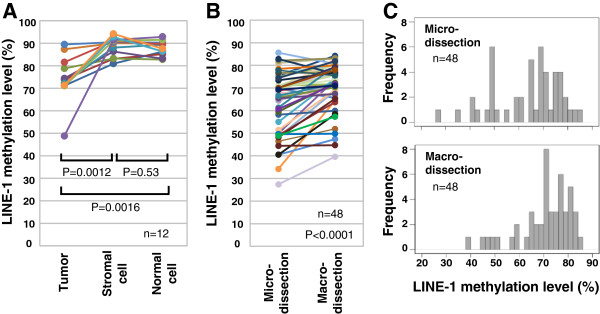
**Influence of stromal cell contamination of the tumor sample on results from the LINE-1 methylation assay. (A)** LINE-1 methylation levels in tumor, stromal and normal cells. **(B)** LINE-1 methylation levels in microdissected tumor cells and in macrodissected tumor tissues. **(C)** Distribution of LINE-1 methylation levels in microdissected tumor cells (upper panel) and in macrodissected tumor tissues (lower panel).

Cancer cells and surrounding stromal cells and normal mucosal cells were obtained from 12 primary tumors and matched adjacent colonic mucosa using LCM. Consistent with a previous report [13], LINE-1 methylation level was significantly lower in tumor cells compared to stromal and adjacent normal cells (Figure 2A). This finding suggests that a high content of stromal cells could influence the accuracy of LINE-1 methylation measurement in tumor samples. To investigate this, we compared microdissected and macrodissected tumor samples, with the latter having moderate-heavy contamination of stromal cells. LINE-1 methylation was significantly lower in the microdissected samples compared to the macrodissected samples (P<0.0001, Figure 2B), with the former also showing more cases with low levels of methylation (Figure 2C).

### Associations between LINE-1 methylation and clinicopathological characteristics

Amongst the 48 primary CRC, 3 were CIMP-positive/microsatellite stable, 1 was CIMP-positive/MSI and 1 was CIMP-negative/MSI. These phenotypes show high LINE-1 methylation [19,20] and distinct clinicopathological features [21,22] and were therefore excluded from the analysis. Table 2 summarizes the associations between LINE-1 methylation in primary tumors and the clinicopathological characteristics of CIMP-negative/microsatellite stable CRC. No significant associations were found, although this could be due to the small sample size. Consistent with a previous report [13], LINE-1 methylation level was lower in the CRC with advanced stage.

**Table 2:** Associations between LINE-1 methylation in primary CRC and clinicopathological features

		**n**	**LINE-1 methylation**	**p-value**
Age	>70	22	69.5 (62.1 – 70.7)	0.17
	≤70	21	59.5 (48.4 – 73.4)	
Gender	male	25	64.4 (48.9 – 70.4)	0.49
	female	18	68.3 (52.3 – 74.3)	
Site	proximal	19	65.3 (53.2 – 69.8)	0.77
	distal	24	69.3 (49.0 – 73.8)	
Histology	well	16	68.6 (49.0 – 72.1)	0.98
	moderately	27	65.7 (54.8 – 71.9)	
T factor	1	1	76.3	0.55
	2	7	67.3 (49.3 – 70.7)	
	3	26	67.3 (49.3 – 70.7)	
	4	9	58.2 (48.2 – 69.3)	
N factor	0	20	69.5 (49.7 – 75.0)	0.67
	1	16	59.4 (48.9 – 74.1)	
	2	7	65.7 (62.9 – 68.0)	
M factor	0	34	67.3 (50.3 – 73.8)	0.49
	1	9	60.7 (48.2 – 70.0)	
Stage	I	4	72.3 (69.5 – 75.0)	0.47
	II	15	69.3 (49.4 – 73.8)	
	III	15	64.4 (50.5 – 68.1)	
	IV	9	60.7 (48.2 – 70.0)	

n, number of patients.

LINE-1 methylation levels are shown as the median (25^th^ percentile – 75^th^ percentile).

Histology of adenocarcinoma was sub-classified into well- and moderately-differentiated adenocarcinoma according to their grading.

TNM factors and stage were defined according to the International Union Against Cancer (UICC) TNM system.

## Discussion

**Figure 3:**
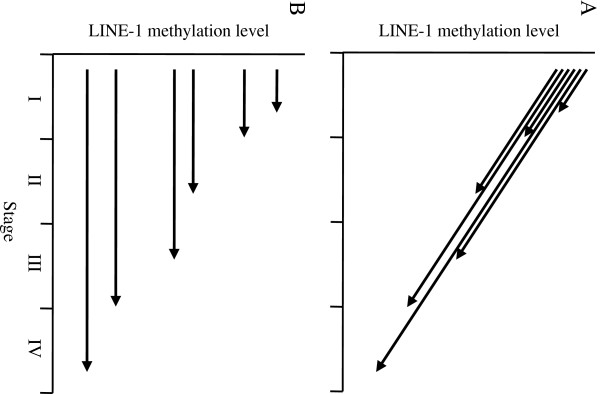
**Possible models to explain the association between LINE-1 hypomethylation and CRC progression. (A)** In the prevailing model, LINE-1 methylation decreases progressively during CRC progression. **(B)** In an alternate model, LINE-1 methylation is stable during CRC progression and the time of diagnosis may be the critical factor that determines the observed association between methylation level and disease stage. In both models, the solid line shows a change of LINE-1 methylation during CRC progression and the arrowhead indicates the time of diagnosis, surgery and sample collection.

LINE-1 hypomethylation has been observed in various malignancies and suggested to play a role in carcinogenesis. Although an association between LINE-1 methylation level and disease stage has been reported in leukemia [23], colorectal [13] and lung cancers [24], the underlying mechanism for this association is unknown. Sunami et al. proposed the concept of a progressive loss of genomic methylation during CRC development [13], as illustrated in Figure 3A. This concept involves that acquisition of metastatic potential in CRC is accompanied by decrease of LINE-1 methylation. From this, it could be expected that LINE-1 methylation level would be lower in metastatic CRC tissue compared to the primary tumor of the same patient if metastasis occurs by a clone of cancer cell with lower LINE-1 methylation. However, our results showed almost identical LINE-1 methylation levels between primary and metastatic sites (both lymph node and distant). Our observation may represent that LINE-1 methylation level is identical between primary tumor cells and metastatic clone, and cannot exclude the concept illustrated in Figure 3A. Nevertheless, it might be unlikely that the LINE-1 methylation is progressively decreased with same grade even in these different cells that reside in different environment including primary, lymph node and distant metastatic sites.

We propose another explanation for the association of LINE-1 methylation with disease stage in Figure 3B. This concept is consistent with the observation that LINE-1 methylation is relatively stable during disease progression and takes into account the time of diagnosis. Cancers with low LINE-1 methylation levels can progress rapidly because they have unstable genomes, more frequent transposition and may be difficult to diagnose early. Such a case is represented by the long arrow in Figure 3B. If the CRC arises within a polyp that has high LINE-1 methylation, such as a serrated adenoma/polyp [25,26], this type of cancer is more likely to be detected early by colonoscopy. In contrast, if the CRC arises within a flat or depressed type of tumor with low LINE-1 methylation, it will be much harder to detect by colonoscopy. Further investigations into LINE-1 methylation and cancer progression are required to test this concept. Possible associations between LINE-1 methylation and the morphology of early stage CRC should also be the subjects of further studies.

LINE-1 methylation is an ideal molecular marker because it exhibits little intra-tumoral heterogeneity. Molecular analysis is usually performed on a small portion of the surgical specimen of primary tumor. In the neoadjuvant setting, tumor tissue obtained by preoperative endoscopic biopsy is used instead of the surgical sample. Intra-tumoral genetic heterogeneity for K-ras and p53 gene mutations has been demonstrated in CRC tissue [27,28]. DNA methylation may show greater heterogeneity than gene mutations because it is a reversible modification. Tumor heterogeneity in samples used for molecular analysis can give rise to false positives and negatives that lead to diagnostic and therapeutic failure. Only one study has so far addressed the issue of intra-tumoral heterogeneity for LINE-1 methylation [13]. This work reported no significant difference in methylation between samples from the luminal surface and those from the most invasive primary tumor site, which is consistent with our data showing no significant difference in methylation between the central area and invasive front from the same tumor. However, there was a weak difference of less than 10% in the LINE-1 methylation level between three analyzed areas, suggesting that some CRCs might have weak heterogeneity in the LINE-1 methylation level. Although further study is required to demonstrate how stable the LINE-1 methylation is in a primary tumor, sampling errors due to strong intra-tumoral heterogeneity are not the case for this molecular marker.

Because the present work found that LINE-1 methylation level was identical between matching primary and metastatic tumor tissues, the molecular analysis of this marker can be performed using tissue samples from either of these sites. Secondary tumor tissues are not readily accessible and hence the molecular features of a metastatic tumor must sometimes be evaluated using the primary tumor, although the reverse is sometimes also the case. The LINE-1 methylation level of an individual CRC patient can therefore be represented by a single sample from any site. One limitation of our work is that cases of metachronous metastases were not included in the CRC study cohort. Considering the long natural history of CRC development, the primary tumor and synchronous metastases evaluated here were from the same time period and hence this could account for the identical levels of LINE-1 methylation. Lower LINE-1 methylation due to clonal evolution might be observed in metachronous metastases. Furthermore, discordance in the LINE-1 methylation level between primary tumor and metachronous metastases might arise in recurrent CRC following chemotherapy. Molecular analysis of relapsed cancer is generally performed on the primary tumor tissue because of the difficulty of access to recurrent cancer sites. Future studies should compare primary tumors and metachronous metastases from individual patients.

In order to use LINE-1 methylation for routine clinical application, LCM is necessary to avoid erroneous results caused by stromal cell contamination. The present study is the first to compare the results for LINE-1 methylation between samples prepared with and without microdissection. LINE-1 methylation level was significantly lower in microdissected compared to macrodissected samples (Figure 2B), demonstrating that measurement of this marker may be inaccurate in tumor samples that have not undergone LCM enrichment. Continuous variables are often stratified into a small number of groups. Absolute assay values are therefore not critical provided their distribution shows a similar pattern between micro- and macro-dissected samples and patients are stratified according to this pattern. However, we observed a considerable alteration in the distribution of LINE-1 methylation values due to contamination with stromal cells (Figure 2C), making it more difficult to achieve accurate stratification of patients. We therefore recommend LCM of tumor samples in order to achieve accurate quantification of LINE-1 methylation.

The multicolor MethyLight assay developed in this study allows the analysis of LINE-1 methylation even in the small amounts of DNA obtained from microdissected samples. Although LINE-1 consists of many numbers of variants [9,29], our MethyLight assay only measures the one of variants, L1.2 (M80343). This major variant of LINE-1 sequence presents in human genome with about 16000 copies compared to single copy gene of ACTB, which is estimated by qPCR and ddCt method (data not shown). The high copy number suggests that the L1.2 sequence can be used to estimate global methylation status. In addition, there was significant relationship between the LINE-1 methylation level measured by this MethyLight assay and those measured by previously developed qMSP assay [18] (Additional file 6: Figure S4). The latter assay was not compatible to microdissected samples but used in our previous studies to demonstrate clinical significance of LINE-1 methylation [15,24]. Together, the new MethyLight assay could be used in broader clinical appreciation than the qMSP assay and in further validation studies of clinical significance of the LINE-1 methylation.

## Conclusions

We have demonstrated that LINE-1 methylation level shows little intra-patient heterogeneity, thus making it suitable for possible clinical applications in CRC. LINE-1 methylation appears to be relatively stable during CRC progression. We propose a new concept to explain the relation between LINE-1 methylation and disease stage. Further studies are required to establish whether LINE-1 methylation could be used in personalized medicine for CRC and to better understand its role in cancer progression and metastasis.

## Competing interests

There are no conflicts of interest with this article.

## Authors’ contributions

AM and KK formulated the design and concept of the study, carried out the methylation assay, and drafted the manuscript. MK, MK and HK participated in DNA preparation, CIMP and MSI analysis. AM and AO carried out preparation of FFPE sections and LCM. GW and TM collected clinical samples and helped to draft the manuscript. All authors read and approved the final manuscript.

## Pre-publication history

The pre-publication history for this paper can be accessed here:

http://www.biomedcentral.com/1471-2407/12/574/prepub

## Supplementary Material

Additional file 1 **Figure S1.** Representative images of tissue sections taken before and after LCM. Click here for file

Additional file 2 **Figure S2.** Methods for the synthesis of assay standards. Click here for file

Additional file 3 **Figure S3.** Accuracy of newly developed LINE-1 MethyLight assay. Click here for file

Additional file 4 **Table S1.** Results of four independent assays for LINE-1 methylation. Click here for file

Additional file 5 **Table S2.** LINE-1 methylation levels in primary tumor of CRC patients. Samples are obtained from tumor center, invasive front and other area. Click here for file

Additional file 6 **Figure S4.** Relationship between the LINE-1 methylation level measured by MethyLight assay and those measured by qMSP assay. Click here for file
