# Supplementary figures and images for: LINE-1 methylation shows little intra-patient heterogeneity in primary and synchronous metastatic colorectal cancer
Source: BMC Cancer. 2012 Dec 5;12:574. doi: 10.1186/1471-2407-12-574 (PMC3534591; doi:10.1186/1471-2407-12-574)

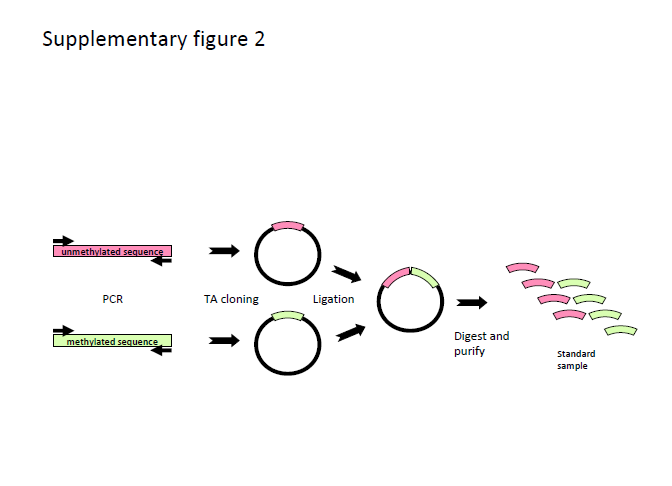

Supplement: Additional file 2 — Figure S2. Methods for the synthesis of assay standards. [file 1471-2407-12-574-S2.doc]

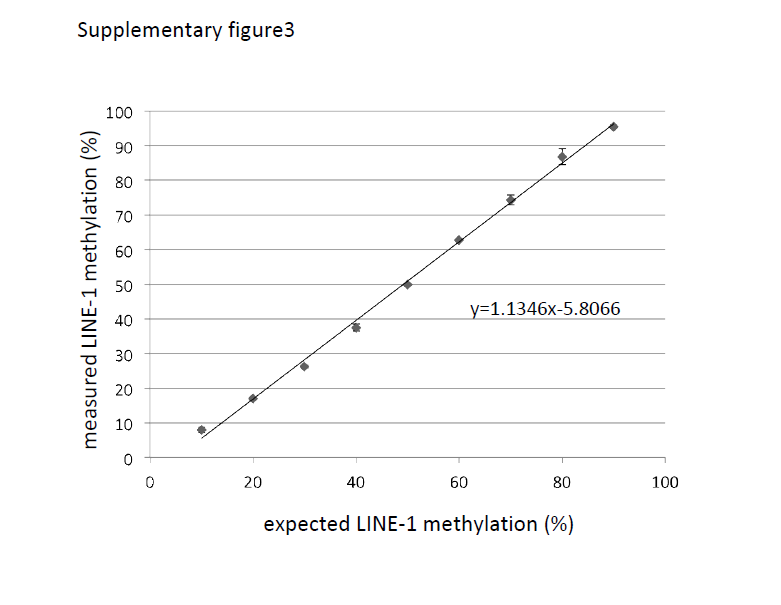

Supplement: Additional file 3 — Figure S3. Accuracy of newly developed LINE-1 MethyLight assay. [file 1471-2407-12-574-S3.doc]

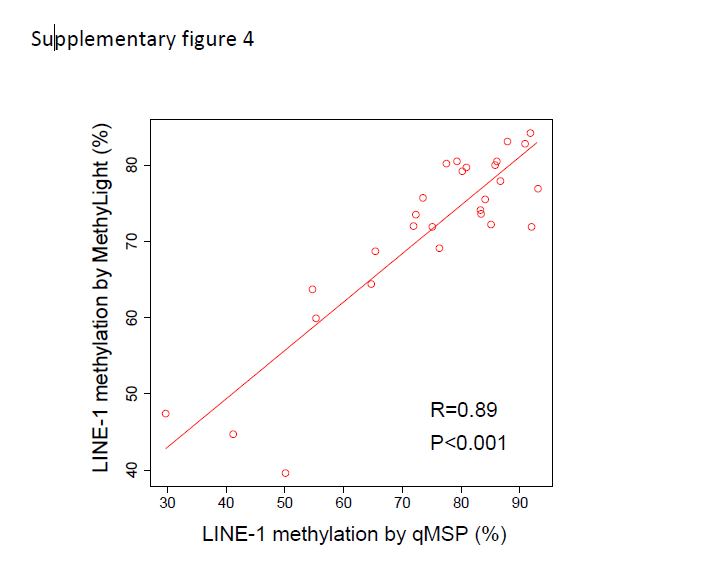

Supplement: Additional file 6 — Figure S4. Relationship between the LINE-1 methylation level measured by MethyLight assay and those measured by qMSP assay. [file 1471-2407-12-574-S6.doc]
