# Supplementary material for: LINE-1 methylation shows little intra-patient heterogeneity in primary and synchronous metastatic colorectal cancer
Source: BMC Cancer. 2012 Dec 5;12:574. doi: 10.1186/1471-2407-12-574 (PMC3534591; doi:10.1186/1471-2407-12-574)

Supplementary figure 1

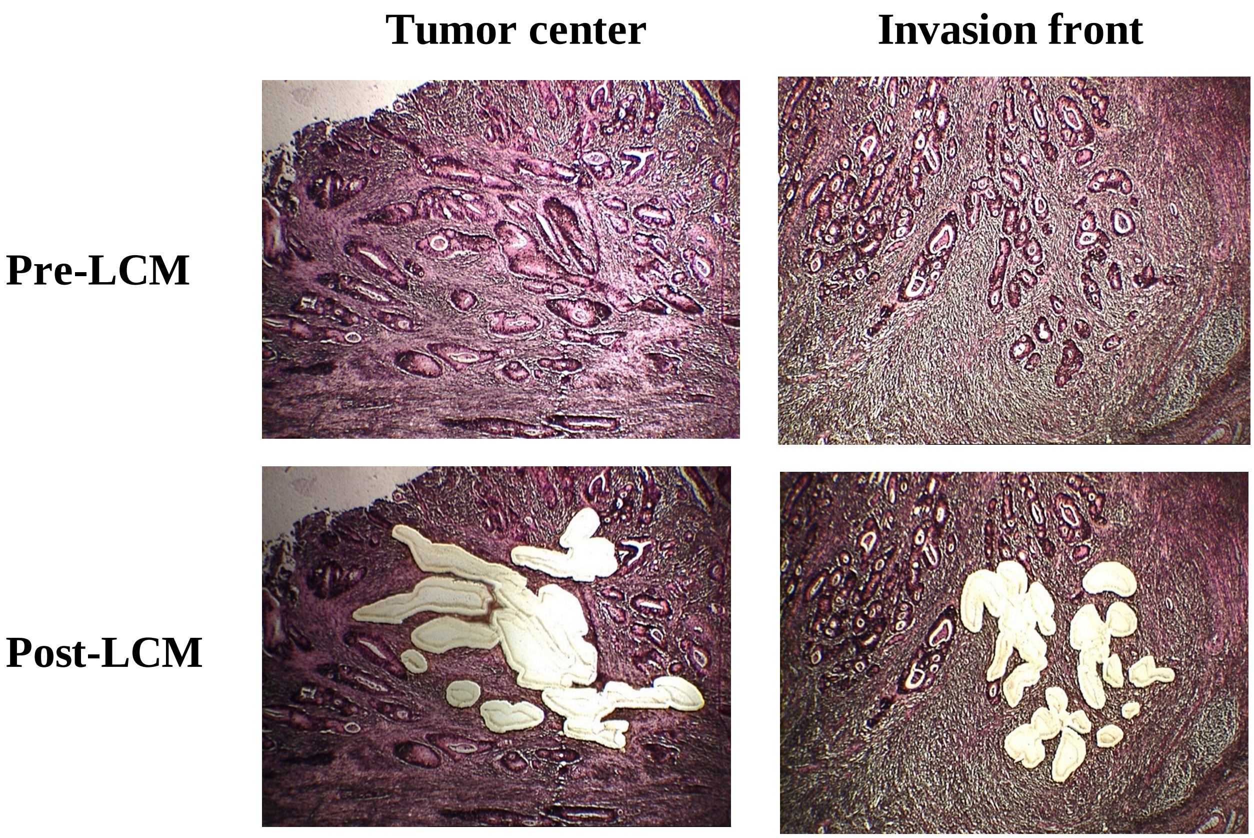

Supplement: Supplementary file 2 [file 1471-2407-12-574-S1.doc]
